# Adjuvant PD-1 Checkpoint Inhibition in Early Cutaneous Melanoma: Immunological Mode of Action and the Role of Ultraviolet Radiation

**DOI:** 10.3390/cancers16081461

**Published:** 2024-04-11

**Authors:** Matthias Brandlmaier, Magdalena Hoellwerth, Peter Koelblinger, Roland Lang, Andrea Harrer

**Affiliations:** 1 Department of Dermatology and Allergology, Paracelsus Medical University, 5020 Salzburg, Austria; m.brandlmaier@salk.at (M.B.); m.hoellwerth@salk.at (M.H.); p.koelblinger@salk.at (P.K.); 2 Department of Neurology, Christian Doppler University Hospital, Paracelsus Medical University and Center for Cognitive Neuroscience, 5020 Salzburg, Austria

**Keywords:** melanomagenesis, PD-1 checkpoint inhibition, UVR exposure, ultraviolet radiation, immune surveillance, skin, lymph nodes

## Abstract

**Simple Summary:**

Melanoma is a type of skin cancer that often spreads and is a significant cause of skin-tumor-related deaths. Checkpoint inhibition with anti-programmed death protein-1 (PD-1) antibodies has significantly improved outcomes for patients with advanced melanoma; however, in the adjuvant setting, not everyone benefits equally. Little is known about the exact underlying immunological mechanisms contributing to the efficacy of anti-PD-1 therapy in patients with completely resected melanoma. This review summarizes the current knowledge on mechanisms of cellular response to adjuvant PD-1 checkpoint inhibition and highlights a possible involvement of ultraviolet radiation.

**Abstract:**

Melanoma ranks as the fifth most common solid cancer in adults worldwide and is responsible for a significant proportion of skin-tumor-related deaths. The advent of immune checkpoint inhibition with anti-programmed death protein-1 (PD-1) antibodies has revolutionized the adjuvant treatment of high-risk, completely resected stage III/IV melanoma. However, not all patients benefit equally. Current strategies for improving outcomes involve adjuvant treatment in earlier disease stages (IIB/C) as well as perioperative treatment approaches. Interfering with T-cell exhaustion to counteract cancer immune evasion and the immunogenic nature of melanoma is key for anti-PD-1 effectiveness. Yet, the biological rationale for the efficacy of adjuvant treatment in clinically tumor-free patients remains to be fully elucidated. High-dose intermittent sun exposure (sunburn) is a well-known primary risk factor for melanomagenesis. Also, ultraviolet radiation (UVR)-induced immunosuppression may impair anti-cancer immune surveillance. In this review, we summarize the current knowledge about adjuvant anti-PD-1 blockade, including a characterization of the main cell types most likely responsible for its efficacy. In conclusion, we propose that local and systemic immunosuppression, to some extent UVR-mediated, can be restored by adjuvant anti-PD-1 therapy, consequently boosting anti-melanoma immune surveillance and the elimination of residual melanoma cell clones.

## 1. Introduction

Melanoma is a malignant skin cancer with a high tendency for metastases. It is the fifth most common solid cancer type in adults worldwide after breast, lung, colorectal and prostate cancer and is responsible for approximately 90% of all skin-tumor-related deaths [1,2]. The most prominent risk factors for melanoma include solar ultraviolet radiation (UVR), a family history of melanoma, high numbers of (dysplastic) nevi, male sex, high age and immunosuppression [3,4,5]. Exposure to UVR is estimated to be responsible for the development of approximately 65% of all melanomas [6,7] and is also the primary modifiable cause. Primary prevention strategies hence aim at protecting the skin to avoid sunburn [8]. 

Whereas stage 0 (in situ, i.e., confined to the epidermis) melanomas usually are cured by excision, this is less frequently the case in stage I and II melanoma. Early stage I and II melanomas already invade the dermis and implicate a considerable risk of recurrence of 2% to 8% in stage IA/B, with one-third recurring as distant metastases even after complete melanoma resection [9,10]. Patients with stage IIB/C melanoma furthermore have a greater risk of recurrence and melanoma-specific death than those with stage IIIA disease [11,12]. For patients with melanoma metastasizing to distant organs, the dramatically dropped median 5-year survival has been raised to about 50% in those receiving immune-checkpoint inhibition (ICI) combination therapy with nivolumab and ipilimumab [13]. 

Thus, avoiding disease progression and melanoma recurrence is of major importance and the main driver towards earlier intervention with ICI therapy. In line is the very recent approval for adjuvant anti-programmed death protein-1 (PD-1) treatment of stage IIB/C melanoma in 2022/2023 [14,15,16] subsequent to the European Medicines Agency approval of the anti-PD-1-inhibitors nivolumab and pembrolizumab for adjuvant treatment of higher-risk stage III melanoma in 2018. 

A main explanation for PD-1 blocking efficacy is that melanoma is a highly immunogenic tumor usually eliciting pronounced cytotoxic tumor-specific T-cell responses [17,18] PD-1 is a co-inhibitory immune checkpoint and strongly expressed on exhausted cytotoxic T cells in settings of chronic inflammation, e.g., the tumor microenvironment (TME). By upregulating programmed death protein ligand-1 (PDL-1), melanoma cells hijack this physiological negative feedback mechanism leading to PD-1/PDL-1-mediated T-cell inactivation and dysfunction for immune evasion [19]. Therapeutic blockade of PD-1 proposedly leads to the reactivation of tumor-specific cytotoxic CD8^+^ T cells, thus reactivating the anti-tumor immune response [20]. 

However, we do not yet understand the underlying mechanism of adjuvant anti-PD-1 therapy, specifically considering the fact that the—supposedly immunogenic—primary tumor mass is lacking. The rationale for earlier therapeutic interventions hence is based on previously demonstrated treatment efficacy in more advanced disease stages rather than on biology [15]. In this review, we aim to delineate a molecular explanation for the efficacy of adjuvant anti-PD-1 therapy and propose two scenarios: boosting of anti-melanoma immune surveillance for eliminating newly transforming pre-malignant melanocytes and the elimination of residual melanoma clones/residual subclinical melanoma in UVR-damaged skin and UVR-immune-compromised skin, respectively. To provide a comprehensive frame on the research gap in the field, we first summarize current landmark trials of adjuvant anti-PD-1 therapy before we delve into what is currently known about the identity of the cells that actually respond to anti-PD-1 therapy and the multifaceted impact of UVR on melanomagenesis, anti-cancer immune surveillance and PD-1 blockade.

## 2. Adjuvant Anti-PD-1 Therapy: Efficacy and Potential Drawbacks

The approval of nivolumab for adjuvant treatment of stage III melanoma was based on data from the CheckMate-238 trial, in which patients with resected stage IIIB, IIIC or IV melanoma (AJCC 7th edition) received one year of adjuvant treatment with either nivolumab at a dose of 3 mg/kg or ipilimumab at 10 mg/kg. After 18 months of follow-up, nivolumab showed superior efficacy compared to ipilimumab. The primary outcome measure was recurrence-free survival (RFS) with a hazard ratio (HR) of 0.65 [97.56% CI, 0.51 to 0.83, *p* < 0.01] [14], further confirmed after 4 years of follow-up (HR 0.71 [95% CI, 0.60 to 0.86, *p* = 0.003]) [21]. In the absence of head-to-head evidence, an indirect comparison of nivolumab (CheckMate 238) versus a placebo (EORTC 18071 trial: ipilimumab vs. placebo) also showed a clinically meaningful reduced relative risk of relapse after 4 years of follow-up with an HR of 0.53 [95% CI, 0.42 to 0.68] in patients receiving adjuvant nivolumab [22]. 

Similar results were obtained with pembrolizumab. In the Keynote-054 (EORTC 1325) trial, patients with resected stage IIIA (at least one lymph node metastasis > 1 mm), IIIB or IIIC melanoma (AJCC 7th edition) either received a fixed dose of adjuvant pembrolizumab (200 mg) every 3 weeks for 1 year or a placebo. RFS was significantly improved in the pembrolizumab arm compared to the placebo with an HR of 0.56 [95% CI, 0.47 to 0.68, *p* < 0.001] after 3 years [23], which was sustained after 5 years of follow-up (HR 0.61 [95% CI, 0.51 to 0.72]) [24]. Also, the toxicity profile of adjuvant pembrolizumab (14.7% grade 3–5 treatment-related adverse events (TRAEs)) [25] was comparable to that of adjuvant nivolumab in the CheckMate-238 trial (14.4% grade 3–5 TRAEs) [14]. 

According to the eighth edition of the AJCC staging system, patients with stage IIB melanoma face a worse prognosis than patients with stage IIIA disease, whereas the prognosis of IIC patients is similar to that for stage IIIB [26]. These findings led to the evaluation of adjuvant nivolumab and pembrolizumab in completely resected stage IIB/C melanoma. The CheckMate-76K trial compared 480 mg nivolumab every four weeks for 1 year to a placebo in 790 stage IIB/C patients, demonstrating a significant improvement in RFS in patients receiving adjuvant nivolumab (HR 0.42 [95% CI, 0.30–0.59, *p* < 0.0001]) [16]. A significant improvement in RFS (HR 0.62 [95% CI, 0.45–0.94]) was also reported for adjuvant pembrolizumab in the Keynote-716 trial, in which 976 patients with resected stage IIB and IIC melanoma received either 200 mg pembrolizumab or a placebo every 3 weeks for 1 year [15]. Details of the trials are summarized in Table 1.

Despite the efficacy of adjuvant anti-PD-1 treatment, there is still a considerable risk of recurrence. After only one year, recurrence rates in adjuvant phase III trials were 37% in stage IV, 25–28% in stage III and approximately 10% in stage II, further increasing to 45–50% in stage III and IV after 5 years of follow-up [14,15,16,24,25,27].

In a retrospective multicenter evaluation, the median time to recurrence in stage III and IV disease patients under adjuvant anti-PD-1-therapy was 4.6 months [95% CI 0.3–35.7]. The majority (76%) of patients experienced recurrence during adjuvant anti-PD-1 treatment indicating an intrinsic, primary resistance, and the remaining 24% experienced recurrence after treatment termination (median of 12.5 months) [28]. These findings are in line with the suggested definition of the Society for Immunotherapy of Cancer of primary resistance/early relapse in response to PD-1 pathway blockade as recurrence at <12 weeks and late relapse as recurrence at >12 weeks after the termination of therapy [29].

Unfortunately, the patterns of disease recurrence are not reported in a standardized way across adjuvant trials; however, initial recurrences most frequently occur at distant sites. This was seen in stage IIB/IIC disease within the Keynote-716 trial, with 47% of all recurrences evolving at distant sites at 27 months [30]. After 5 years of follow-up, distant recurrence rates in patients with stage III/IV disease were 57% in the CheckMate-238 and 63% in the Keynote-054 trial [24,27]. Real-world data confirm these recurrence patterns, with 57% of stage III/IV melanoma recurrences happening distantly [28]. Also, the development of new primary melanoma has been reported in 1–2% of patients undergoing adjuvant anti-PD-1 therapy [15,27].

The prevention of disease recurrence is still the main driver of dynamically evolving perioperative and/or combination treatment strategies.

The combination of anti-PD-1 therapy with other immune checkpoint inhibitors has been successful in the metastatic setting and is currently being evaluated in the adjuvant setting. Ongoing adjuvant phase III trials examine dual checkpoint inhibition with nivolumab and the LAG-3 inhibitor relatlimab in completely resected stage III disease in the Relativity-098 trial (NCT05418972), the combination of cemiplimab (anti-PD-1) and fianlimab (anti-LAG-3) in patients with stage IIC-IV melanoma (NCT05608291), and the co-formulation of pembrolizumab and vibostolimab (inhibitor of T-cell immunoreceptor with Ig and ITIM domains (TIGIT)) in resected stage II–IV disease in the Keyvibe-010 trial (NCT05665595). 

However, in the CheckMate-915 trial, the addition of 1 mg/kg ipilimumab every 6 weeks to 240 mg nivolumab every 2 weeks did not achieve a clear RFS benefit in comparison with 480 mg nivolumab every four weeks, but was associated with substantially increased toxicity (33% vs. 12.8% grade 3–5 TRAEs) [31] (see Table 1). Of note, the schedule of ipilimumab administration in this trial was different from that routinely used in the advanced disease setting. 

In general, the risk of severe acute as well as long-term toxicities is another potential drawback of adjuvant anti-PD-1 immunotherapy, which must be particularly considered in patients with relatively low risk of recurrence (e.g., stage IIB, III A) [32].

**Table 1:** Pivotal phase III trials involving adjuvant anti-PD-1 immunotherapy in resectable high-risk melanoma; * AJCC 7th edition; ^#^ AJCC 8th edition; Abbreviations: CLND, complete lymph node dissection; RFS, relapse-free survival; TRAEs, treatment-related adverse events.

Trial	Stage	Trial Arms (n Patients)	CLND	RFS (%)	Grade 3–5 TRAEs (%)
**CheckMate-238 [14,21,27,33,34]**	IIIB, IIIC, IV *	Nivolumab vs. Ipilimumab A: 3 mg/kg nivolumab; Q2W (n = 453) B: ipilimumab 10 mg/kg Q3W 4x, followed by Q12W ≤1 year (n = 453)	Yes	Nivolumab (Arm A) −12 months: 70.5% −24 months: 62.6% −36 months: 58.0% −48 months: 51.7% −60 months: 50.0%	Nivolumab (Arm A): 14.4%
**Keynote-054 [23,24,25]**	IIIA- IIIC *	Pembrolizumab vs. Placebo A: 200 mg pembrolizumab Q3W, 18 doses (~1 year) (n = 514) B: placebo (n = 505)	Yes	Pembrolizumab (Arm A) −12 months: 75.4% −24 months: 68.3% −36 months: 63.7% −60 months: 55.4%	Pembrolizumab (Arm A): 14.7%
**SWOG S1404 [35]**	IIIA- IIID, IV *	Pembrolizumab vs. Adjuvant Standard of Care A: 200 mg pembrolizumab Q3W for 1 year (n = 647) B: high-dose IFNα-2b or 10 mg/kg ipilimumab Q3W 4x, followed by up to 11 doses Q12W (n = 654)	Yes	Not reported	Pembrolizumab (Arm A): 19.5%
**CheckMate-915 [31]**	IIIB- IIID, IV ^#^	Nivolumab/Ipilimumab vs. Nivolumab alone A: 240 mg nivolumab Q2W + ipilimumab 1 mg/kg Q6W (n = 920) B: 480 mg nivolumab Q4W (n = 924)	Yes	Nivolumab/Ipilimumab (Arm A) −24 months: 64.6% Nivolumab Arm (Arm B) −24 months: 63.2%	Nivolumab/Ipilimumab (Arm A): 33.0% Nivolumab Arm (Arm B): 12.8%
**CheckMate-76K [16]**	IIB- IIC ^#^	Nivolumab vs. Placebo A: 480 mg nivolumab Q4W (n = 526) B: placebo (n = 264)	No	Nivolumab (Arm A) −12 months: 89%	Nivolumab (Arm A): 10.5%
**Keynote-716 [15,36]**	IIB- IIC ^#^	Pembrolizumab vs. Placebo A: 200 mg pembrolizumab Q3W, 17 cycles (n = 487) B: placebo (n = 489)	No	Pembrolizumab (Arm A) −12 months: 90% −18 months: 86% −36 months: 76%	Pembrolizumab (Arm A): 17%

## 3. Mechanisms of Cellular Response

A clinically detectable melanoma has escaped from the cancer–immune equilibrium, a phase during which subclinical melanoma already has been initiated but the immune system is in control and prevents further growth and spread [17]. The underlying mechanisms of melanoma immune evasion are reasonably well understood and include downregulation of tumor-associated antigens (e.g., MART-1, tyrosinase, and gp100, cancer testis antigens, neoantigens), upregulation of co-inhibitory receptor ligands such as PDL-1 [17,37], downregulation of MHC-I-class molecules [17,38,39,40] and a plethora of further mechanisms contributing to a tumor-promoting microenvironment [17]. 

Upregulation of PDL-1 and downregulation of MHC-I-class molecules are particularly relevant, as these two mechanisms complement each other, allowing melanoma cells to evade MHC-I-restricted killing by tumor-specific cytotoxic CD8^+^ T cells and instead render them dysfunctional via the PD-1/PDL-1 pathway. Interfering with CD8^+^ T-cell dysfunction by immune checkpoint blockade has become a therapeutic cornerstone in advanced melanoma. The proposed concept behind treatment with anti-PD-1 antibodies was to reinvigorate dysfunctional or exhausted CD8^+^ T cells within the TME to counteract tumor progression [41,42]. 

In the case of adjuvant anti-PD-1 therapy in completely resected stage II/III melanoma, the biology is less clear, because the tumor mass is lacking. Furthermore, the biology behind T-cell dysfunction is complex, and the question of which cells mediate anti-PD-1 and adjuvant anti-PD-1 therapy responses remains. The term T-cell dysfunction has been coined for cancer immunology in analogy to T-cell exhaustion in chronic infection. In chronic infection, pathogens persist, and sterilizing immunity cannot be achieved. The chronically stimulated antigen-specific T cells gradually loose effector functions and concomitantly upregulate inhibitory receptors such as PD-1 [43,44]. In contrast to acute infection, cancers develop slowly through clonal evolution [45], during which they either initially evade T-cell recognition simply by lack of sufficient cancer antigens required for activating antigen-presenting cells or by suboptimal priming of tumor-specific T-cell responses in tumor-draining lymph nodes (TDLNs) [46]. Subsequent accumulation of mutations and increased expression/release of tumor antigens during cancer transformation may promote increased antigen presentation in TDLNs and increased infiltration of the tumor with tumor-reactive T cells. From there on, a continuous encounter with persistent tumor antigens (rather than microenvironmental factors such as PDL-1 upregulation) drives T-cell dysfunction, possibly explaining the incongruent results regarding PDL-1 expression in tumors and response to anti-PD-1 therapy [47]. The dysfunctional differentiation program is triggered during tumor initiation, epigenetically imprinted and, importantly, reversible only at early stages [17,47]. During this dynamic process, T cells progressively loose effector functions such as the ability to proliferate, produce cytokines and kill target cells [46,48,49]. Early and late tumor-specific dysfunctional T cells have equally impaired cytotoxicity and express PD-1 and LAG-3. Late dysfunctional T cells can be distinguished from early dysfunctional T cells by the expression of additional inhibitory receptors such as CD38, CD39, CD101 and TIM3 [47,50]. 

The fact that distinct states of T-cell dysfunction exist strongly suggests that exhausted T cells from early to late dysfunctional states cannot be equally well boosted or respond equally well to PD-1 blockade. Indeed, early dysfunctional T cells have been increasingly described to be the least epigenetically constrained, with reversible dysfunction similar to the so-called progenitor-exhausted T cells from chronic infection models [47,51,52,53]. Also, there is no evidence that dysfunctional CD8^+^ T cells within tumors are able to regain function or expand in response to PD-1 blockade. Instead, increased proliferation of circulating lymphocytes and clonotypic expansion of T cells observed in the blood were associated with superior clinical responses [54,55]. Other studies demonstrated a clonal replacement of exhausted intratumoral CD8^+^ T cells with non-exhausted T cells from outside the tumor during anti-PD-1 therapy [56]. 

Importantly, tumor-specific CD8^+^ T cells readily identified in TDLNs are generally of an early dysfunctional phenotype and express intermediate levels of PD-1 and the chemokine receptor CXCR5 and high levels of T-cell factor 1 (TCF-1) [52,54]. Moreover, these cells are capable of a proliferative burst in response to PD-1 blockade and highlight the TDLNs as a reservoir for CD8^+^ T cells responsive to anti-PD-1 therapy [57,58,59,60,61].

Collectively, these studies propose that early dysfunctional tumor-specific PD-1^+^ CD8^+^ T cells in sites of micrometastases, lymph nodes and blood may be the actual target cells of therapeutic anti-PD-1 antibodies, as they expand, infiltrate the tumor and most likely also engage in the anti-tumor immune defense [46,62]. Moreover, early dysfunctional tumor-specific CD8^+^ T cells circulating in blood implicate their systemic distribution, migration into neighboring but also more distant lymph nodes and homing to the site of the skin lesion and beyond. Early dysfunctional melanoma-specific CD8^+^ T cells accordingly are still present, even after complete resection of the primary melanoma and affected TDLNs. Hence, they are also plausible target cells in the setting of adjuvant PD-1 blocking therapy.

## 4. The Multifaceted Effects of UVR (in Melanomagenesis)

Susceptibility to developing a clinically evident melanoma may arise from repeated UVR-induced skin stress, as cutaneous melanoma has been consistently associated with a history of repeated sunburns [63,64,65,66]. In fact, 85% of cutaneous melanomas present evidence of UVR signature mutations in their genomes [18,67]. The association between sun exposure and melanomagenesis, however, is complex because some melanomas arise at sun-shielded anatomical sites or without known etiological association with UVR exposure [68]. Here, inherited transcriptional programs involved in melanocyte positional identity, microenvironmental permissive niche cues and thyroid hormone effects, among others, may control melanoma biology [68,69].

The dual pathway hypothesis, first proposed by Holman et al. [70] and based on observations of intermittent versus continuous patterns of sun exposure promotes two distinct biological pathways by which cutaneous melanoma might develop. Accordingly, it differentiates between a globally estimated 75% of cutaneous melanomas associated with cumulative sun damage arising from excess sun exposure at sites such as head and neck and those arising on less frequently sun-exposed sites in people with many nevi [71,72,73]. In other words, melanomas arising from chronically sun-damaged skin (i.e., head and neck) proposedly evolve from distinct evolutionary trajectories compared to those arising from less frequently UVR-exposed sites [74,75]. 

Still another layer of complexity arises from the fact that melanomas are not a direct consequence of UVR-exposure-associated damage but usually develop later during the course of life [69]. This highlights the importance of revisiting the broad immunomodulatory properties of UVR on top of its direct skin-damaging effects, which might hinder proper immune responses to the neoplastic transformation of melanocytes in the long term. UVR-mediated effects on adaptive immune responses are subtle consequences of the initial innate immune responses and the associated inflammatory milieu [76,77]. They can occur locally and systemically, and their net effects are immunosuppressive, most likely to avoid immune-mediated further destruction instead of the resolution of the UVR-induced damage and inflammation. 

In the following, we outline current knowledge about the significance of UVR-induced skin stress from sunburn to systemic immunomodulation. This serves as a knowledge base as to why adjuvant anti-PD-1 therapy can effectively enhance immune surveillance against cancer.

### 4.1. Direct and Indirect Effects of UVR on the Skin and the Sunburn Response 

The main mechanisms by which UVR induces skin tumorigenesis involve direct effects on DNA mutations and indirect effects via the promotion of inflammation and suppression of anti-tumor immune responses [75].

Solar UVR contains 6% UVB radiation (waveband 280–320 nm) capable of penetrating only as far as the epidermis and 94% UVA radiation (waveband 320–400 nm) penetrating deeper into the dermis [78]. Both are considered phototoxic because they elicit the production of reactive oxygen species resulting in oxidative stress [79,80,81]. The small fraction of shorter-wavelength UVB radiation is particularly relevant for skin cancer development because it exerts both direct and indirect detrimental UVR effects on the skin [78]. Direct UVR-induced damage, in particular, the formation of cyclobutane pyrimidine dimers from the interaction of UVB with DNA [82], can give rise to cancer-driving mutations in oncogenes and tumor suppressor genes if it remains unrepaired [75,76]. 

**Figure 1:**
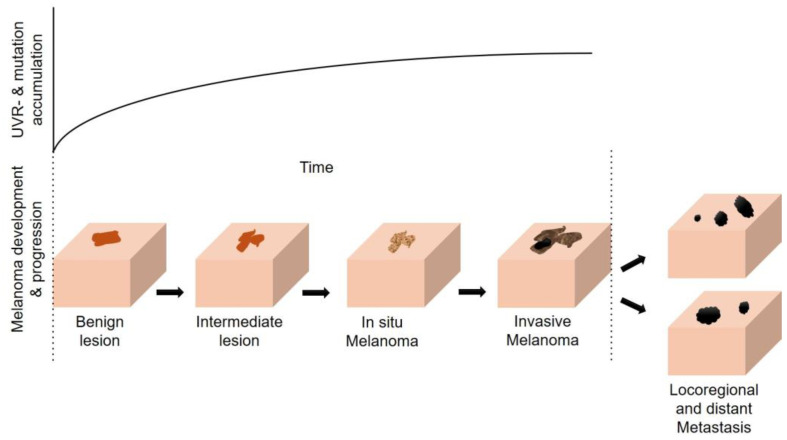
Model for melanoma progression during UV exposure (adapted from [84]).

The relevance of direct UVR-induced DNA damage for melanomagenesis is highlighted by the fact that melanocytes contain over 2000 genomic sites that are extraordinarily sensitive to UVR, which perfectly align with recurrent UV signature mutations (characteristically C > T and CC > TT transitions) in individual gene promoters of melanomas and known cancer drivers [83]. UVR thus has been implicated as a major factor in both the initiation and progression of melanoma since it increases genomic instability in melanocytes (Figure 1) [84]. Interestingly in a murine model, the ability to initiate melanoma formation after UVR exposure was higher in NRAS-mutant melanocytes as compared to BRAF-mutant melanocytes, whereas mutational burden was higher in BRAF-driven tumors [85]. A growing body of evidence suggests that modifications in the epigenetic landscape associated with UVR exposure drive the alteration of transcriptional programs that are tightly associated with the development of melanoma [86,87].

Dose and intensity certainly are major factors determining the initial impact of UVR. Normal UVR exposure causes keratinocytes to release alpha-melanocyte stimulating hormone (α-MSH), which signals melanocytes that there is a need for melanin. The binding of α-MSH to the melanocortin-1 receptor (MC1R) initiates the transcription of microphthalmia-associated transcription factor and the synthesis of tyrosinase, tyrosinase-related protein 1 and pre-melanosome protein [79,88,89,90,91]. Melanocytes hence react with increased melanogenesis and the transfer of UVR-absorbing melanin granules to keratinocytes for protecting their nuclear DNA like a sun cap [92,93]. The resulting tanning protects both keratinocytes and melanocytes themselves from repeated modest UV irradiation [69,94,95,96]. In addition, MC1R is a key regulator of melanogenesis and determines the skin tone of tanning by controlling the relative proportions of eumelanin and pheomelanin [69]. Critically, MC1R also is the most polymorphic gene in the human genome [69]. Many natural human MC1R variants occur and the carriers of MC1R variants, in particular those with pale skin, freckles and red hair, have an around 60% higher risk of developing melanoma [97,98,99,100]. Data from a B16F10 mouse melanoma model suggest that increased activity of MC1R as result of UVR exposure dampens the anti-tumor T-cell response [101].

In the case of excessive solar UVR exposure leading to sunburn, the situation regarding melanogenesis is completely different. Sunburn manifests as erythema, increased blood flow, mast-cell degranulation and skin peeling associated with keratinocyte cell death, increasing the risk of melanoma by exposing melanocytes to direct UVR damage and UV-induced gene mutations [77].

The sunburn response furthermore is characterized by acute self-resolving inflammation, triggered by the release of damage-associated patterns (DAMPs) such as self-RNAs, oxidized lipids, heat shock proteins and high-mobility group box 1 protein (HMGB1) released from UVR-damaged and necrotic keratinocytes [76]. The binding of DAMPs to Toll-like receptors on intact neighboring cells activates downstream type I interferon responses and the release of TNFα, IL-6, IL-10 and IL-1b [75,76,102], which again contributes to neutrophil granulocyte, monocyte and T-cell infiltration of the skin.

Both acute inflammation and transition into the resolving phase are mainly determined by indirect UVR effects through the absorption of UVB photons by chromophores in membrane lipids, proteins and nuclear DNA of epidermal cells. The resultant UVB-induced products include reactive oxygen species but also potent immune mediators such as platelet-activating factor (PAF), PAF-like molecules, prostaglandin E, cytokines (e.g., TNFα, IL-4, IL-6, IL-10, IL-33), antimicrobial peptides, cis-urocanic acid and the active pre-vitamin D3 metabolite 1.25 (OH)_2_ D_3_.

These immune mediators directly or indirectly activate cells in the epidermis and dermis, recruit circulating immune cells and, importantly, instigate immune suppression for the resolution of the acute innate inflammation [76,102].

### 4.2. UVR Effects on Innate and Adaptive Immune Cells

**Figure 2:**
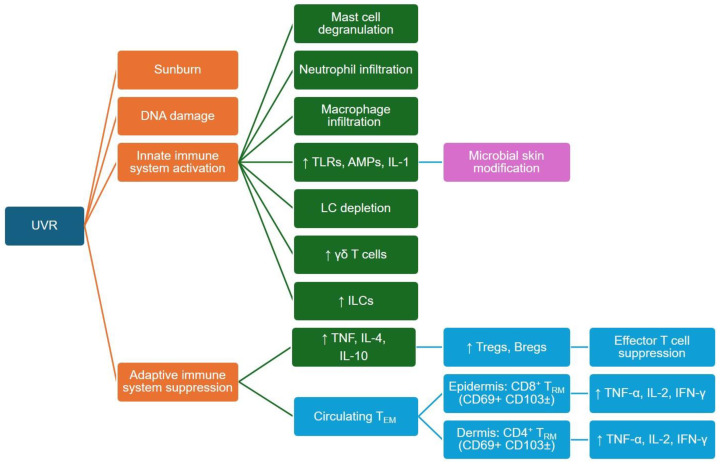
UVR initiates a cascade of skin responses, color-coded here to indicate processes and outcomes: DNA damage and sunburn (orange), activation of innate immune cells like macrophages and mast cells (green) and induction of immune suppression via Tregs and B regs leading to inhibition of effector T cells (blue). The commensal microbiome’s influence on cytokine, AMP and TLR generation is also depicted (purple). The dynamics of T_RM_ cells and their cytokine profiles in various skin layers are highlighted, emphasizing the nuanced interplay between skin immunity and UVR. The precise impact of UVR on these T_RM_ cell populations, however, remains to be uncovered. Adapted from [103]. Abbreviations: AMP, antimicrobial peptide; Bregs, regulatory B cells; IFN, interferon; IL, interleukin; ILC, innate lymphoid cell; LC, Langerhans cell; T_EM_, effector memory T cells; TLR, Toll-like receptor; TNF, tumor necrosis factor; Tregs, regulatory T cells; T_RM_, tissue-resident memory T cells; UVR, ultraviolet radiation; ↑, increase.

Understanding and integrating these extremely pleiotropic and dose-dependent indirect UVR effects, each dampening or interfering with specific aspects of the immune response, is challenging. In the following paragraphs, we summarize published evidence (visualized in Figure 2) on how UVR may modulate the innate and adaptive immune crosstalk in the skin, which, mainly based on in vivo animal and in vitro cell culture data, provides important mechanistic clues regarding a compromised anti-cancer immune surveillance but might not fully represent human biology.

Langerhans cells (LCs) are professional antigen-presenting cells that reside in the epidermis, where they continuously sample the microenvironment. Upon activation, they migrate to the skin-draining lymph nodes to prime and activate adaptive effector cell responses. The respective effector functions depend on the activation state of LCs. Exposing LCs in vitro to UVR leads to a depressed antigen processing and presenting function [104,105,106] and downregulated expression of costimulatory CD80 and CD86 [107], resulting in impaired interactions with adaptive immune cells.

**Figure 3:**
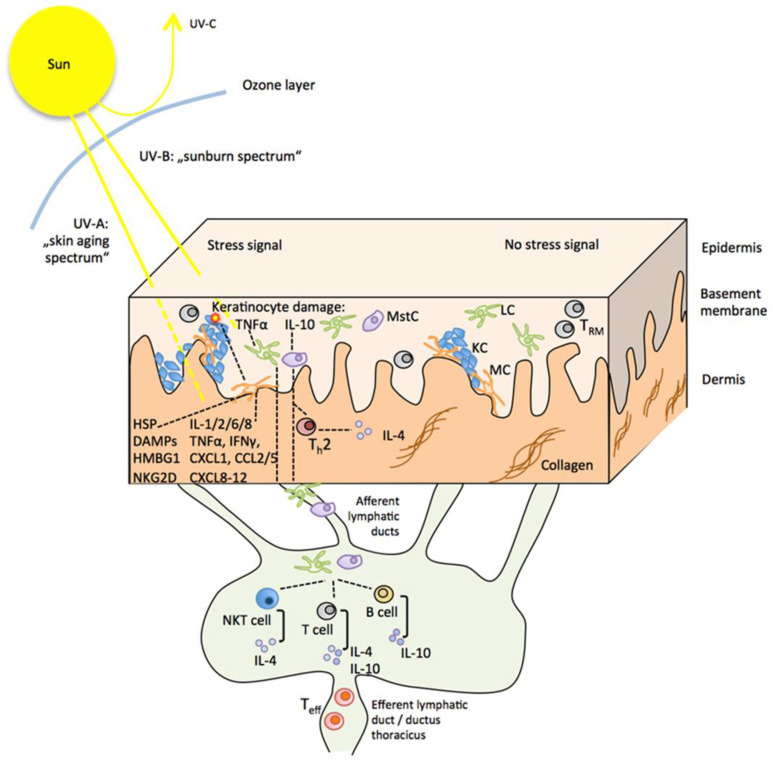
Sun exposure and stress signaling in the skin and its draining lymph nodes. Under normal conditions, a homeostatic equilibrium is maintained in the skin without stress signals (right part of the figure). Solar UVB (290–320 nm) and UVA (320–400 nm) radiation (left part of the figure), capable of penetrating the epidermis (UVB) and dermis (UVA), cause a stress response from skin cells, initiating direct local inflammation and indirectly promoting an immunosuppressive environment. This includes an innate response promoted by the production of pro-inflammatory cytokines and danger-associated molecular patterns from keratinocytes and melanocytes and the activation of tolerogenic LCs via the release of anti-inflammatory IL-10 from keratinocytes and mast cells. Upon migration to the skin-draining lymph nodes, tolerogenic LCs prime regulatory immune cell phenotypes in T and B cells, which travel to the skin where they suppress adaptive immune surveillance. Abbreviations: LC: Langerhans cell, MstC: mast cell, IL: interleukin, IFNγ: interferon gamma, TNFα: tumor necrosis factor alpha, CCL: CC-chemokine ligand, CXCL: chemokine (C-X-C motif) ligand, HSP: heat shock protein, DAMPs: damage-associated molecular pattern molecules, HMBG1: high mobility group protein B1, NKG2D: NKG2-D type II integral membrane protein, Teff: effector T cell, T_RM_: skin-resident memory T cells.

In mice, anti-inflammatory IL-4, IL-10 and TGFβ released from UVR-exposed keratinocytes, skin-infiltrating neutrophils and mast cells promote a tolerogenic phenotype in LCs. Upon migration into skin-draining lymph nodes, they prime regulatory rather than effector phenotypes in CD4^+^ T and B cells and immunosuppressive natural killer T cells, which may release IL-4 and IL-10, contributing to systemic immune suppression or migrate to the exposed skin, contributing to local immune suppression [76,77,102,108,109,110,111,112,113,114,115] (summarized in Figure 3). In addition, fewer antigen-specific effector and memory T cells are generated, which insufficiently migrate to the site of UVR exposure [76].

IL-10 released from UVR-exposed keratinocytes and mast cells furthermore promotes a local Th2 T-cell response and secretion of IL-4 in the dermis [116]. Mast cells also directly transmit immunosuppressive UVR responses from the skin to the lymph nodes. Constitutively expressing CXC-chemokine receptor 4, they follow cognate CXC-chemokine ligand 12 gradients into B cell follicles where mast-cell-derived IL-10 negatively affects T follicular helper function and antibody production [108,111,117], and they contribute to the formation of IL-10-producing B cells (summarized in Figure 3) [76,108].

Indirect UVR effects on adaptive immune cells further involve reduced activity of IL-17-producing tissue-resident memory T (T_RM_) cells with dampened recall responses and suppressed functions of CD4^+^ and CD8^+^ effector T cells [118,119].

Interestingly, natural killer (NK) cells apparently are the sole immune cell subset not suppressed by UVR [120]. NK cells were found to be recruited into the epidermis probably regulated by TNF-alpha released from UVR-activated LCs [121]. Data from melanoma research furthermore revealed that crosstalk of activated NK cells with conventional DC-type 1 cells even may mitigate UVR-induced immunosuppression on melanoma-specific T cells [108,122,123]. 

### 4.3. UVR Effects on the Skin’s Neuroendocrine System

A frequently neglected but increasingly recognized topic is that the UVR-associated photoproducts also involve the release of mediators such as neurotransmitters, neuropeptides and hormones. Particularly interesting examples are neuropeptides including pituitary (proopiomelanocortin-derived adrenocorticotropic hormone (ACTH), beta-endorphin or MSH peptides, and thyroid-stimulating hormone (TSH)) and hypothalamic (corticotropin-releasing factor (CRF) and related urocortins, thyroid-releasing hormone (TRH)) hormones, which are hierarchically produced and organized as hypothalamic–pituitary–adrenal axis (HPA) and hypothalamic–thyroid axis (HPT) analogs in the skin [124]. 

The activation or modification of cutaneous HPA elements depends on higher-energetic UVB wavelengths and results in fine-tuning and selective regulation of skin pigmentation as well as innate and adaptive skin immunity, among other results [124]. The physiological role of molecular elements of the HPT axis such as functional TSH and TRH receptors and biologically active TSH and TRH expressed on keratinocytes, melanocytes and dysplastic nevi is less clear [124]. UVR-damaged keratinocytes, however, are suspected to be involved in thyroid autoimmune diseases by triggering anti-TSH receptor antibodies [125], whereas TRH upregulation is proposedly involved in the malignant conversion of melanocytes into melanoma cells and their further proliferation and progression [126]. 

## 5. Adjuvant Anti-PD-1 Therapy—Boosting Anti-Cancer Immune Surveillance 

### 5.1. Elimination of Newly Transforming Pre-Malignant Melanocytes

UVR, hence, has been proposed as a complete carcinogen in melanomagenesis, exhibiting both direct (DNA damage) and indirect tumor-promoting effects by impairing immunosurveillance of (pre-)malignant transforming melanocytes [75]. Fortunately, during the course of life, human skin usually tolerates repeated acute intermittent UVR exposures and also sunburns without developing a clinically evident melanoma, suggesting sufficient immune activity capable of monitoring and eliminating (pre-)malignant transforming melanocytes in most cases. 

One reason may be that melanocytes themselves display considerable immune reactivity in response to oxidative stress, such as that caused by sunburn. Similar to keratinocytes, melanocyte stress responses include the production of heat shock proteins and DAMPs; the upregulation of Toll-like receptors, NKG2D ligands (e.g., MICA/B and ULBP1), and PDL-1 [108]; the release of pro-inflammatory cytokines and chemokines; and the upregulation of adhesion and costimulatory receptors [127]. The signaling pathways that are activated determine the outcome, namely DNA repair, cell cycle arrest or cell death, and depend on genetic, intracellular and intercellular interactions [82]. The location of melanocytes at the dermo-epidermal junction furthermore facilitates their interaction with infiltrating immune cells, and the expression of NKG2D ligands makes them vulnerable to immune-targeted destruction by UVR-resistant cytotoxic NK cells [120,128]; altogether, these mechanisms preclude the malignant transformation of severely stressed cells during acute UVR-induced inflammation. 

An alternative scenario involves dysfunctional melanocytes, which do not self-destruct or become apoptotic but trigger stromal remodeling, local tissue disruption, rapid recognition of these danger signals and an innate anti-tumor response involving NK cells and LCs as professional antigen-presenting cells, among others [17,129]. Cytotoxic NK cells exert immune-targeted destruction of NKG2D-, NKp30-, NKp46- or DNAM-1-overexpressing transformed cells [130]. Activated LCs ingest and process the resultant cell debris; migrate to the draining lymph nodes; and prime melanocyte/melanoma-specific cytotoxic CD8^+^ T cells, CD4^+^ helper T cells and B cells. These cells can migrate back to the tumor site and drive secondary adaptive responses, primarily MHC-I-restricted CD8^+^ T cell-mediated cytotoxicity and likely antibody-dependent cellular cytotoxicity.

However, melanoma can eventually develop, which may be attributed to melanoma-intrinsic factors, failing skin immunity, or both. Direct DNA-mutating UVR effects contributing to melanomagenesis are well established, whereas evidence linking UVR exposure to defective immune surveillance is limited [75,76,82,131]. 

An important contribution is the work of Hawkshaw et al., who provided human in vivo data demonstrating that only a single pro-inflammatory exposure to UVR leads to lasting immunosuppressive effects involving regulatory CD8^+^ GATA3^+^ T cells and an elevated prostaglandin synthesis [132]. They proposed the prevention of chronic inflammation and autoimmunity against the considerable release of self-antigens during acute and self-resolving sunburn response as the biological rationale of the prolonged post-resolution immune suppression [132]. UVR-damaged melanocytes, however, may remain silent for many years, until proliferative cues arise and instigate their malignant transformation or formation of nevi [133]. Whether any acute UVR-associated post-resolution immune suppression in the skin may last and contribute to a melanoma-enabling tissue environment after several years, hence, remains to be shown. 

Beyond acting locally in the skin, potent, chromophore-derived immune mediators also trigger a cascade of systemic immune effects [80,102]. These proposedly include cellular changes in draining lymph nodes, hematopoietic alterations in the bone marrow and modulations in the composition of circulating blood cells [80], which may contribute to the beneficial effects of sunlight or phototherapy in some systemic autoimmune diseases [80,102] but at the same time may compromise immune surveillance. Chromophore-derived mediators released into the circulation also include neuropeptides and hormones of the cutaneous HPA. These may communicate UVR-induced skin-derived messages leading to activation of the central HPA with cortisol/corticosterone serving as a final effector towards systemic immunosuppression [124]. 

Still another aspect is that sunburns have been denoted a clinical phenotype of photoaging, as are increased wrinkles, epidermal atrophy, increased tanning and irregular pigmentation [134], and that melanoma is most common in older men with an average age of 65 at diagnosis, hence physiologically aged skin [135]. Both photoaging and chronological aging involve the accumulation of senescent skin cells with keratinocytes and melanocytes displaying a pro-inflammatory phenotype termed senescence-associated secretory phenotype (SASP) [136,137]. In SASP, a chronic low-grade inflammation prevails, which stimulates a counteracting expansion of immunosuppressive cells (e.g., Treg, myeloid-derived suppressor cells, regulatory DCs) and production of anti-inflammatory cytokines (e.g., IL-10, TGF-beta) [138,139].

Taken together, the combined effects of a local post-sunburn-resolution immune suppression and/or UVR-associated systemic immunosuppression on top of a (photo)aging-induced remodeled skin immune system provide a permissive environment for melanomagenesis. The boosting of pre-existent anti-melanoma immune surveillance through adjuvant anti-PD-1 therapy may be effective in the immediate elimination of (pre-)malignant transforming melanocytes in patients with resected primary tumors.

### 5.2. Elimination of Residual Melanoma Clones/Subclinical Melanoma

Increased risk of melanoma recurrence after complete resection derives from a primary melanoma in the vertical growth phase that has already invaded the dermis (stage IIB/IIC) and possibly metastasized to skin-draining lymph nodes (stage IIIA–D) [10,115]. For clinically visible melanomas, there is a fundamental possibility that tumor remnants or individual tumor cells were not removed by the complete surgical resection and that individual clones may have already migrated loco-regionally, into draining or more distant lymph nodes as well as other organs.

Since residual melanoma clones have already escaped immune control once, they seem likely to be able to escape again due to their malignant potential. Moreover, the remaining melanoma-specific cytotoxic CD8^+^ T-cell repertoire assumedly is in a dysfunctional state, and continued life-long UVR exposure may further increase the risk as a predominant mutagen through all melanoma stages towards malignant neoplasm [74]. 

Residual melanoma hence may not be successfully eliminated by any local de novo forming immune response or by any pre-existing melanoma-specific immune surveillance through CD4^+^ and CD8^+^ memory T and B cells generated during an encounter with the primary tumor [17]. It is furthermore questionable whether such clones or subclinical melanomas can be contained by the adaptive immune defense in a so-called cancer–immune equilibrium [17]. Even melanoma-specific CD8^+^ T_RM_ cells may not be able to contain them, although their fundamental role in suppressing subclinical melanoma progression recently was identified in a mouse model of transplanted melanoma [140].

Preventive reactivation of existing melanoma-specific cytotoxic T cell memory through adjuvant anti-PD-1 therapy appears reasonable considering the malignant properties of residual melanoma clones in frequently photoaged skin and that further sun exposures involving UVR-induced immune suppression, as outlined above, is orchestrated through the crosstalk among immune cells within the skin microenvironment and draining lymph nodes (illustrated in Figure 3) [37]. Accordingly, UVR exposure mediating local and systemic immune suppression implicates local and systemic tumor-growth-promoting environments in skin and/or lymph nodes for residual melanoma cells. This is supported by a recent observation that UVR stress can induce early PD-L1 expression in melanocytes and melanoma cells by a DAMP- and HMGB1-mediated pathway independent from IFN-gamma-associated immune evasion [37]. Further exposure to the sun, hence, may promote the evasion of residual melanoma cells from the pre-existing tumor immune surveillance. Such melanoma-specific CD8^+^ T-cell memory is present in the skin, the regional draining lymph nodes of the resected site and the blood [141,142,143,144,145]. It also includes early dysfunctional tumor-specific PD-1^+^ CD8^+^ T cells in lymph nodes and blood proposed as bona fide responders to PD-1/PD-L1 blockade [42,46]. Systemic reactivation and expansion of melanoma-specific, anti-PD-1-responsive CD8^+^ T cells and their infiltration of the skin may also be capable of complete elimination or at least of controlling the growth of residual melanoma cells in a UVR-compromised environment.

The earlier this happens, the more beneficial this may be for the outcome, since UVR significantly contributes to the accumulation of somatic mutations. These may cause the malignant transformation of melanocytes and progression of melanoma, act as neoantigens to elicit anti-tumor immune responses and cause a high tumor mutation burden (TMB), which further increases during melanoma progression [146,147]. Tumors with increased TMB present more neoantigens and, thus, are more immunogenic [148,149,150] and associated with improved response to anti-PD-1 therapy [147,151,152,153,154]. This has been attributed to persistent tumor mutational burden, which tends to be more clonal in melanoma and may drive sustained responses to anti-PD-1 therapy [155]. 

However, tumors with equally high TMB levels but variable immune responses [156], tumors with low TMB levels responding to immunotherapy [153] and the predicted neoantigen load not correlating with T cell infiltration in melanoma [157] fueled the identification of intratumor heterogeneity (ITH) as an additional factor influencing immune surveillance and predicting response to anti-PD-1 therapy [152,158,159,160]. ITH is manifested by the distribution of clonal versus subclonal mutations and neoantigens [157,161], taking into account that in more heterogeneous tumor cell populations, reactive neoantigens may undergo “dilution” relative to other neoantigens, increasing the chance of tumor cells escaping immune surveillance [151]. This is corroborated by TCGA (The Cancer Genome Atlas) melanoma patient data, revealing a significantly higher survival rate with tumors of a fewer number of clones [151]. 

UVR strongly contributes to neoantigen formation and may promote the formation of subclonal mutations and the development of ITH of recurring melanoma. Early interference with melanoma recurrence and ITH formation through adjuvant anti-PD-1 therapy may be particularly beneficial regarding a later-stage treatment scenario, in which checkpoint immunotherapy rather may counter-productively result in selecting for low-ITH tumor cells [162] and allow high-ITH melanoma cells to progress. The relevance of the quality of UVR-induced somatic mutations and neoantigens over their quantity has also been reported by Lo et al. [163], who demonstrated a skewing of epitopes recognized by T cells towards wild-type tumor-lineage self-antigens as an underlying mechanism for successful responses to checkpoint blockade and long-term immunity. 

This may also explain the development of vitiligo, a depigmentation disorder resulting from the autoimmune destruction of melanocytes, among patients with melanoma receiving anti-PD-1 therapy. Vitiligo likely arises from epitope-spreading towards melanocyte-lineage antigens shared by melanocytes and melanoma cells, and its appearance is associated with higher rates of objective responses to anti-PD-1 therapy [164]. Beyond provoking a strong anti-tumor response against UVR-mutation-containing residual melanoma cells, adjuvant reactivation of tumor-specific T cells, hence, may also evoke epitope spreading towards (pigmentation-related) wild-type melanocyte antigens as a pathway for an improved outcome.

## 6. Conclusions 

Anti-PD-1 therapy is effective in melanoma owing to the highly immunogenic nature of this malignancy. Blocking PD-1 is believed to reinvigorate CD8^+^ T cells, hence restoring the impaired anti-tumor immune response. In spite of countless studies investigating the mechanisms of action of PD-1 blockade, several questions remain. This particularly applies to the adjuvant setting, in which the vast majority of the supposedly immunogenic tumor cells (primary tumor, affected lymph node) have been surgically removed. Still, adjuvant immune checkpoint inhibition is clinically beneficial even though the underlying biological mechanisms remain to be elucidated. 

**Figure 4:**
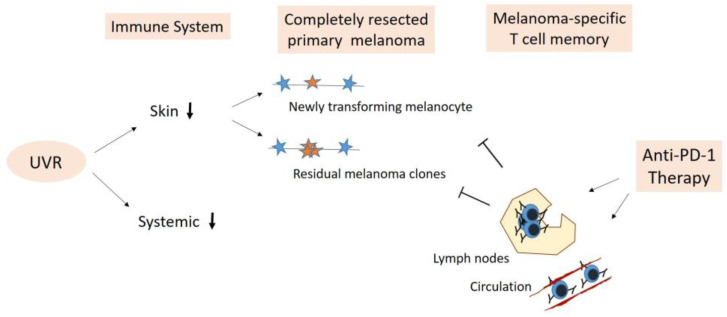
Immunological explanation for the efficacy of early adjuvant anti-PD-1 therapy. UVR as a major risk factor may lastingly compromise skin and systemic immunity in patients with completely resected primary melanoma. Reactivation of pre-existing (early dysfunctional) melanoma-specific CD8 + T-cell memory present in lymph nodes and circulation by anti-PD-1 therapy may boost anti-melanoma immune surveillance for (i) immediate elimination of any newly transforming pre-malignant melanocytes and (ii) successful killing of any residual melanoma clones. ↓, restricted function.

Solar UVR exposure is the most prominent risk factor for primary melanoma development and may also contribute to melanoma recurrence. The multifaceted effects of UVR include direct mutational DNA damage in melanocytes, early upregulation of PD-L1 contributing to immune evasion, compromised regional skin immune surveillance and priming of adaptive anti-cancer immune response as well as systemic immune suppression via the cutaneous neuroendocrine system. Aside from their implications for melanomagenesis, mutational UVR effects are also key to the immunogenicity of melanoma cells. The efficient elimination of these cells by reactivated melanoma-specific T cells and resultant epitope spreading allow for long-term immunity in response to anti-PD-1 therapy. Long-term efficacy is maintained through functional melanoma-specific CD8^+^ memory T cells, which persist in various locations such as skin, lymph nodes and the bloodstream—even after complete resection of the primary tumor and affected TDLNs. Particularly in UVR-compromised conditions, adjuvant anti-PD-1 therapy has the potential to reinvigorate T cell memory, hence boosting anti-melanoma immune surveillance and aiding in the elimination both of de novo transforming and residual melanoma cells (Figure 4).

This potential results in rapidly evolving perisurgical, monotherapy and combination (neo-)adjuvant treatment strategies, all of which still face the major problem of primary checkpoint inhibitor resistance. Therefore, identifying patient subgroups most likely to benefit from (adjuvant) ICI treatment currently has the highest priority in order to avoid over-treatment and limit toxicity while improving individual treatment efficacy.
